# Analysis of Spontaneous Plant Species in an Urban Green Space in Southern Spain

**DOI:** 10.1002/pei3.70144

**Published:** 2026-04-30

**Authors:** María José Tenor‐Ortiz, Purificación Alcázar, Rafael Tamajón‐Gómez, Carmen Galán

**Affiliations:** ^1^ Department of Botany, Ecology and Plant Physiology, Agrifood Campus of International Excellence CeiA3 University of Córdoba Córdoba Spain; ^2^ Andalusian Inter‐University Institute for Earth System IISTA University of Córdoba Córdoba Spain

**Keywords:** allergenicity, climate change, green space management, heat island effect, spontaneous flora, urban biodiversity

## Abstract

This study presents a detailed floristic inventory of the spontaneous flora in La Asomadilla urban park in Córdoba, southern Spain. In addition to being the largest urban park in the city, La Asomadilla is characterized by irregular terrain, a transitional location between the Guadalquivir river plain and Sierra Morena mountains, and the predominance of native vegetation with a naturalized appearance rather than a formal garden design. These characteristics make the park an important reservoir of biodiversity and a barrier against the spread of invasive species. A total of 250 species belonging to 60 families were recorded, predominantly therophytes adapted to Mediterranean climatic conditions. Two species ( *Cyperus eragrostis* and *Valerianella microcarpa*) were recorded for the first time in the municipality, along with five orchid species of high ecological value. Several allergenic taxa were identified, primarily belonging to the Poaceae, Asteraceae, and Oleaceae families, highlighting the importance of considering the impact on public health in the management of urban biodiversity. Unlike most studies on urban green spaces, which focus mainly on ornamental or tree species, this research highlights the relevance of the accompanying wild flora. These species not only contribute to climate change mitigation by reducing temperatures and capturing CO_2_ but also provide health benefits for the population. Therefore, this study provides novel and valuable data at the local level to inform ecological and public health management strategies in Mediterranean urban areas.

## 1 Introduction

Green areas, such as urban parks, gardens, boulevards, and other spaces in the city, play an essential role in creating more sustainable and healthy urban environments (Cariñanos et al. 2016). They are increasingly important spaces due to their influence on people's quality of life as they contribute to improving air quality by reducing airborne pollutants (particulate matter), help mitigate urban heat through temperature regulation, and contribute to climate regulation through carbon dioxide sequestration (Latinopoulos et al. 2016; Granados Espíndola et al. 2022). Thus, providing a place of recreation for society living in urban environments.

However, the limited availability of green urban spaces is primarily the result of urbanization process and land use planning decisions. This reduction in vegetation cover can exacerbate temperature increases in urban areas and intensify urban heat effects. The intensification of heatwaves in the Mediterranean region is a consequence of climate change, and their impacts are further amplified in urban areas due to urbanization process (Ali et al. 2022). One of the most pervasive applications of urban vegetation is the regulation of solar radiation (Ochoa de la Torre 1999; Moreno et al. 2023), with the objective of attenuating the effects of the phenomenon known as the Urban Heat Island (UHI). This phenomenon is evidenced by the difference in temperature between urban and rural areas. This is evidenced by an increase in temperature compared to areas with greater vegetation cover (Barrera Alarcón 2018). The UHI effect is a growing problem, with an impact on human thermal comfort and public health in general, reducing the quality of life in cities (Moreno et al. 2023).

In addition to the UHI effect, urban environments are characterized by elevated levels of pollutants, including Diesel Exhaust Particles (DEP) and their components (D'Amato et al. 2010; Ziello et al. 2012). These particles interact with biological particles, facilitating the transport of aeroallergens that enter the mucous membranes of the respiratory tract and the eyes, causing irritation. The emission of biological Volatile Organic Compounds (VOC) and biological particles from urban vegetation has also the potential to result in episodes of air pollution that may have implications for human health (Cariñanos et al. 2016; Orru et al. 2017). The interaction of pollen with atmospheric pollutants has been observed to result in an increased incidence of allergic reactions (Cabrera et al. 2021; Berger et al. 2021; Fernández‐Rodríguez et al. 2016; Fernández‐González et al. 2023; Mousavi et al. 2024).

The elevated level of carbon dioxide (CO_2_) in urban areas, in conjunction with the increase in temperature, has been demonstrated to result in an increase in flowering, and then in pollen production, attributable to the observed increase in plant biomass (Damialis et al. 2019; Ziska 2021). Several studies have demonstrated that the consequences of climate change, including higher temperatures and increased air pollutants, have the potential to significantly alter the distribution and flowering intensity in allergenic species, such as Poaceae (Ziska et al. 2003; Barber et al. 2008; Ziska et al. 2009; Damialis et al. 2019; Ziska 2021; Cristofolini et al. 2024) with probably serious health consequences.

In this context, it is crucial to emphasize the considerable value of urban flora as a natural filter for pollutants. Recently, there has been a growing interest in native flora, which requires minimal economic maintenance, is adapted to local climatic conditions (Cardona Dahl 2002; Martínez Sánchez et al. 2008; Bayón et al. 2021), and shows resistance to pests and diseases (Reyes et al. 2021).

The introduction of non‐native species into domestic gardens is becoming increasingly prevalent (Herrero‐Borgoñón et al. 2005; Legg and Kabisch 2024), giving rise to concerns about their potential to become invasive in natural habitats (Aymerich 2013; Hu et al. 2023). The implementation of scientific planning and optimization strategies for urban green spaces has the potential to enhance the urban‐ecological aspect (Moreno et al. 2020) and improve ecosystem services (Janhäll 2015). Furthermore, the use of native species in urban green spaces facilitates the conservation of plant diversity and prevents the establishment of invasive species in natural environments. The conservation of biodiversity in urban green spaces promotes diversity in horticulture and ensures the continued existence of wild species (Di Martino et al. 2020; Andrade et al. 2021).

The aim of this study is to develop a floristic catalogue of spontaneous plant species in the urban park “La Asomadilla” in Córdoba, identifying major life forms, chorological affinities, and species with allergenic potential. The study seeks to provide baseline data to support improved urban biodiversity management at the local level, with special focus on ecological and public health considerations.

## 2 Material and Methods

### 2.1 Study Area

The study area chosen is the green space known as “La Asomadilla,” which was selected because it is the largest urban park in the city of Córdoba, and the second largest in Andalusia, where new floral references for the municipality have been documented, providing valuable information for local urban biodiversity management (Devesa 2015; López‐Tirado 2018; Velasco‐Jiménez et al. 2020). Considering the accepted biogeographical system of the Iberian Peninsula (Rivas‐Martínez et al. 1997; Loidi 2017), most of Córdoba municipality is included within Campiña of Guadalquivir Sector (Hispalense Sector) of Baetic Province, specifically in the Low Campiña District. The studied park is placed in the “North Sierra” of the city of Córdoba, transition area between the mentioned sector and the Mariánica Range Sector of the West Iberian Mediterranean Province (Lusitania and Extremadura Subprovince), within the Pedroches and Alcudia District. Therefore, its unique characteristics were crucial in selecting it as a study area, thus constituting an enclave of high ecological relevance. Its location in the transition zone between one unit and another generates great environmental heterogeneity, with irregular topography, naturalized landscapes and a predominance of native species that serve as biodiversity reserves. Furthermore, the park's design, with 27 ha and 1372 trees (Velasco‐Jiménez et al. 2020), eschews the traditional formal garden style in favor of spontaneous vegetation, which increases its ecological value and resilience against invasive species. The city of Córdoba is situated in the south of the Iberian Peninsula and has a Mediterranean‐continental climate. According to the “Andalusian Agroclimatic Information Network” (RIA, in Spanish), this is characterized by mild winters with moderate rainfall and a summer drought with high temperatures. The Andalusian Institute for Agricultural, Fisheries, Food and Ecological Production Research and Training (IFAPA, in Spanish) has indicated that the average annual temperature from 2000 to 2021 was 17.61°C, with an average annual rainfall of 611.58 mm during the same period. After Valle et al. (2004) this city is classified in the bioclimatic belt thermo‐mediterranean and the dry‐subhumid ombrotype.

### 2.2 Working Methodology

The field work has been extended from February to the end of August in 2021, to cover different seasonal phenological periods (winter, spring and summer) of the flora and vegetation. Although autumn sampling was not conducted, winter conditions in the thermo‐Mediterranean bioclimatic belt allow the detection of most geophytic taxa. However, some strictly autumn‐flowering species may have been underrepresented. Prior to the vegetation study, reconnaissance surveys were conducted, during which the species observed were recorded. These surveys were carried out on February 15, March 1, March 15 and April 6. For the identification of the species, the keys of the “Flora Vascular del Término Municipal de Córdoba” (López‐Tirado 2018) were used. Specific bibliography (Subiza Garrido‐Lestache 2004; Cariñanos et al. 2014; Cabrera 2011) was used to identify species with allergenic potential.

Field reconnaissance routes were established in order to gain an understanding of the diversity of environments and microhabitats that exist within the park. Four zones were differentiated:

- Areas strongly managed (141,198 m^2^): lawns, orchard edges, roadsides.
- Natural areas or with low intensity management (10,422 m^2^): bush, pond and surroundings.
- Olive grove (7322 m^2^): lookout point area, north‐eastern part.
- Mediterranean perennial grassland dominated by tall tussock forming grasses (“cerrillar”) (4091 m^2^): southern part of the lookout point.

During the flora and vegetation surveys only wild species were recorded, including both spontaneous and subspontaneous plant species, not considering the ornamental and cultivated ones; however naturalized or escaped individuals growing spontaneously within the study area were recorded when present.

The methodology used has been previously consulted in local floristic studies comparable to our study (López‐Tirado 2018; Eljebri et al. 2024). The floristic catalogue was obtained from the study of plant communities using the phytosociological method (Braun‐Blanquet 1979) as detailed in Table 1. Subsequently, the floristic catalogue was analyzed according to life forms using the classification system proposed by Raunkiaer (1934), based on the location of the perennating bud after unfavorable season (Table 2). Conversely, the species were classified in accordance with their geographical provenance (Table 5), following the chorological criteria established in *Flora Iberica* (Castroviejo 1986) and relevant contributions in *Flora Montiberica (*Alejandre 1989; Escudero 1992; Fernández 1991; Loidi et al. 1997; Navarro Sánchez 1989; Castroviejo 1986), in order to analyze the relationship between chorological origin and life forms.

**TABLE 1:** Abundance‐dominance indices according to the Braun‐Blanquet (1979) scale.

5	Coverage of 75% or more
4	Coverage from 50% to 75%
3	Coverage from 25% to 50%
2	Coverage from 5% to 25%
1	Coverage less than 5%
+	Few or sporadic (2–5 individuals)
r	A single individual, negligible coverage

**TABLE 2:** Raunkiaer life form classification system (Raunkiaer 1934).

Phanerophytes	Buds at least 50 cm above ground
Chamaephytes	Buds up to 50 cm above ground
Hemicryptophytes	Buds above or just below ground
Cryptophytes or geophytes	Buds below ground
Therophytes	Annuals
Hydrophytes	Water plants

**FIGURE 1:**
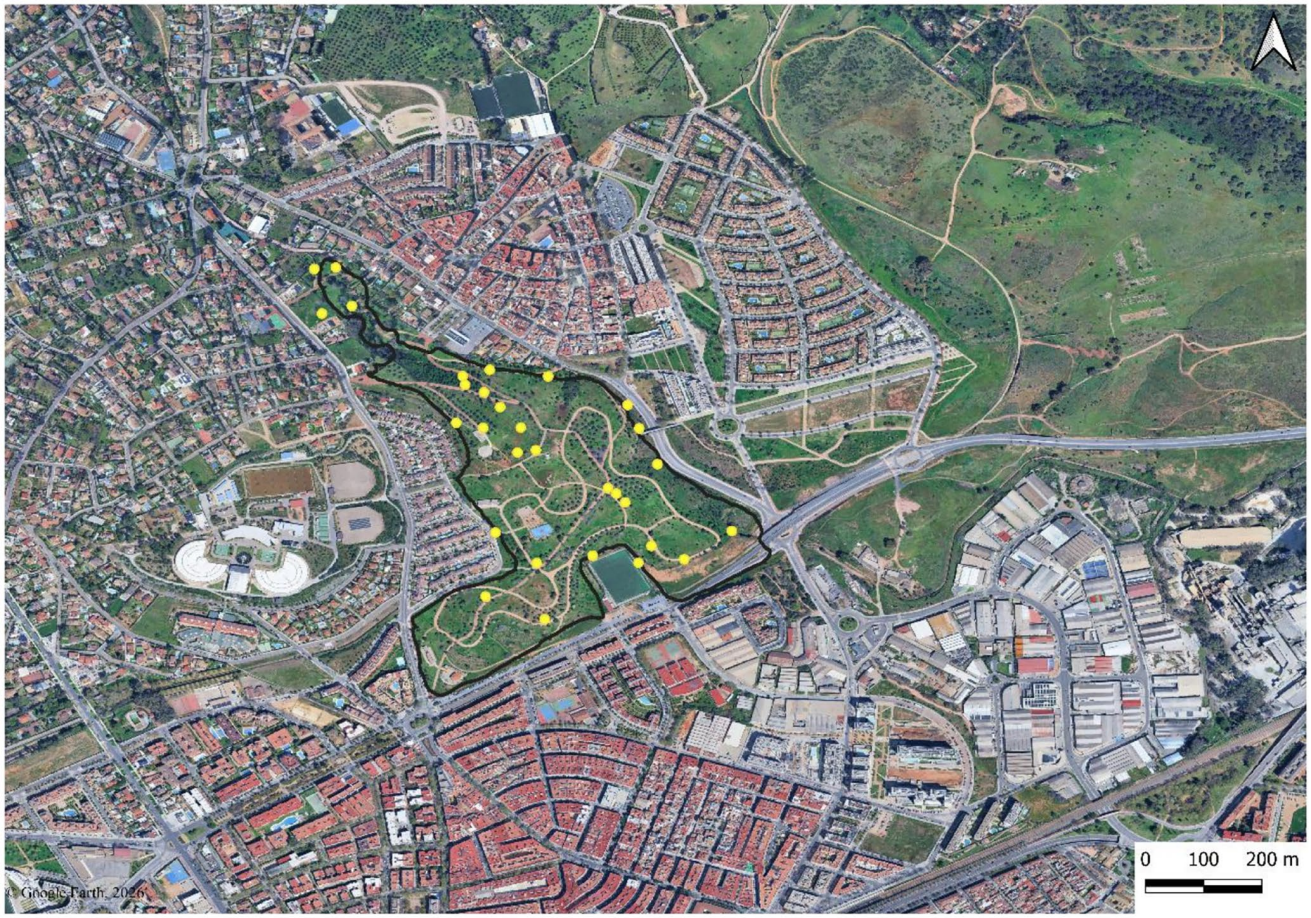
Randomly selected points in “La Asomadilla”. Base map: Google Earth satellite imagery (Google, 2026; accessed January 2026).

A second level in the floristic study entailed the analysis of the most frequent species in the most managed areas, which in this case were the lawns. A sampling scheme was devised with the establishment of 25 randomly selected points using QGIS 3.14.0 (QGIS Development Team 2020) (Figure 1). At each point, within a square of 1 m^2^, the species present were noted, indicating only their presence or absence. This level of floristic analysis focuses exclusively on the most managed area, as this is the most widespread type of habitat in the park.

## 3 Results and Discussion

### 3.1 Floristic Survey

In the study area, 251 species belonging to 60 families have been observed (Table S1). Comparing these results with those obtained by López‐Tirado (2018) at the level of the municipality of Córdoba (1226 species belonging to 158 families), 20.47% of the vascular flora and 37.97% of the families are represented in “La Asomadilla.” Of the total, 27 species are allochthonous, two of which are included in the Spanish Catalogue of Invasive Species: *Arundo donax* L. and *Oxalis pes‐caprae* L. The high number of botanical families in this study is representative of the high biodiversity (Table 3).

**TABLE 3:** Floristic composition: numerical and taxonomic summary.

Category	Value
Total number of species recorded	251
Total number of botanical families	60
Non‐native species	27
Invasive species	2
New records for the municipality	2
Orchid species	5
Main families	Asteraceae (37), Poaceae (28), Fabaceae (26)
Dominant biological form	Therophytes (142 species, 56.57%)
Dominant phytogeographic origin	Mediterranean (66 species, 26.61%)
Species with allergenic potential	Species from Poaceae, Asteraceae, Oleaceae and Amaranthaceae

The most prevalent families are those evolutionary more advanced, with the possibility to be better adapted to aridity, that is, Asteraceae, with 37 species (14.74%), followed by Poaceae with 28 species (11.16%) and Fabaceae with 26 species (10.36%). Lamiaceae and Apiaceae are represented with 10 species each (4%). The remaining families are less represented, with the majority comprising one or two species (Table S1).

The most common species were *Avena sterilis* L., (36%); *Crepis vesicaria* subsp. *taraxacifolia* (Thuill.) Thell. (33%) and *Dactylis glomerata* . subsp. *hipanica* (Roth) Nyman. (31%). The remaining species have a presence of less than 30%.

### 3.2 Life Forms

The recorded species have been classified according to their life forms (Table 4), with six categories defined by Raunkiaer (1934). The majority of species (142) are defined as therophytes, accounting for 56.57% of the total; additionally, one of them is parasitic, *Orobanche nana* (Reut.) Beck (Table 3). Hemicryptophytes account for 20.32% of the total number of species, followed by geophytes with 8.76% representation. These results are consistent with previous works in agrosystems (Chafik et al. 2013; Osman et al. 2014; Eljebri et al. 2024). The predominance of species with these life forms, identified in our study, may be related to the fact that many of these species can easily resprout and expand after disturbances to their environment (Debussche et al. 1996; Santana Pastor 2011).

**TABLE 4:** The life forms of the species recorded in the study area, as classified by Raunkiaer (1934).

Life forms	Number of species	Percentage (%)
Phanerophyte (Ph)	25	9.96
Chamaephytes (Ch)	11	4.38
Hemicryptophytes (He)	51	20.32
Geophytes (Ge)	22	8.76
Therophyte (Th)	142	56.57
Hydrophytes (Hy)	2	0.80

### 3.3 Chorological Affinities

The results of the chorological analysis of the identified species are presented in Table 5. The Mediterranean element is strongly represented, with 66 Mediterranean species, 44 Later.‐ Circum.‐ Mediterranean species, and 35 Euro.‐Circum.‐Med. species. These results coincide with Velasco‐Jiménez et al. (2020), in which they studied different ornamental species in the same study area where the predominant origin was the Mediterranean. On the other hand, many of the species found in the area are cosmopolitan, with *Cynodon dactylon* (L.) Pers, *Daucus carota* L., and *Daucus crinitus* Desf. standing out as the most abundant in this type of phytogeographic region.

**TABLE 5:** Summary of phytogeographical distribution of species uncovered in the study area. The classification has followed the criteria and categories of *Flora Ibérica* and *Flora Montiberica*.

Phytogeographical	Number of species	Percentage (%)
Med	66	26.61
Later.‐Circum.‐Med	44	17.74
Cosm	35	14.11
Euro.‐Circum.‐Med	30	12.10
Holar	25	10.08
Ir.‐Tur	17	6.85
Iber.‐Afr	7	2.82
Sub.‐Med	7	2.82
Paleotrop	6	2.42
Euras	3	1.21
Neotrop	2	0.81
Euro.‐Sib	1	0.40
Eurosib.‐Med.‐Ir.‐Tur	1	0.40
Iber.‐Magre	1	0.40
Med.‐Euras	1	0.40
Med.‐Ir.‐Tur	1	0.40
Med.‐Paleotrop	1	0.40

Abbreviations: *Cosm*, Cosmopolitan; *Euras*, Eurasian; *Euro.‐Circum.‐Med* Euro.‐Circum.‐Mediterranean; *Euro.‐Sib*, Euro‐Siberian; *Eurosib.‐Med.‐Ir.‐Tur*, Euro‐Siberian‐Mediterranean‐Irano‐Tauranien; *Holar*, Holarctic; *Iber.‐Afr*, Ibero‐African; *Iber.‐Magre*, Ibero‐Maghrebi; *Ir.‐Tur*, Irano‐Turanian; *Later.‐Circum.‐Med*, Later.‐Circum.‐Mediterranean; *Med*, Mediterranean; *Med.‐Euras*, Mediterranean‐Eurasian; *Med.‐Ir.‐Tur*, Mediterranean‐Irano‐Turanian; *Med.‐Paleotrop*, Mediterranean‐ Paleotropical; *Neotrop*, Neotropical; *Paleotrop*, Paleotropical; *Sub.‐Med*, Sub Mediterranean.

The high prevalence of therophyte species in the study area reflects their adaptation to the arid and hot Mediterranean climate, particularly the unfavorable summer period, as well as their effective dispersal capacity and ability to thrive under stressful environmental conditions and in modified cropping systems (Lazarina et al. 2019; Irl et al. 2020; Eljebri et al. 2024).

**FIGURE 2:**
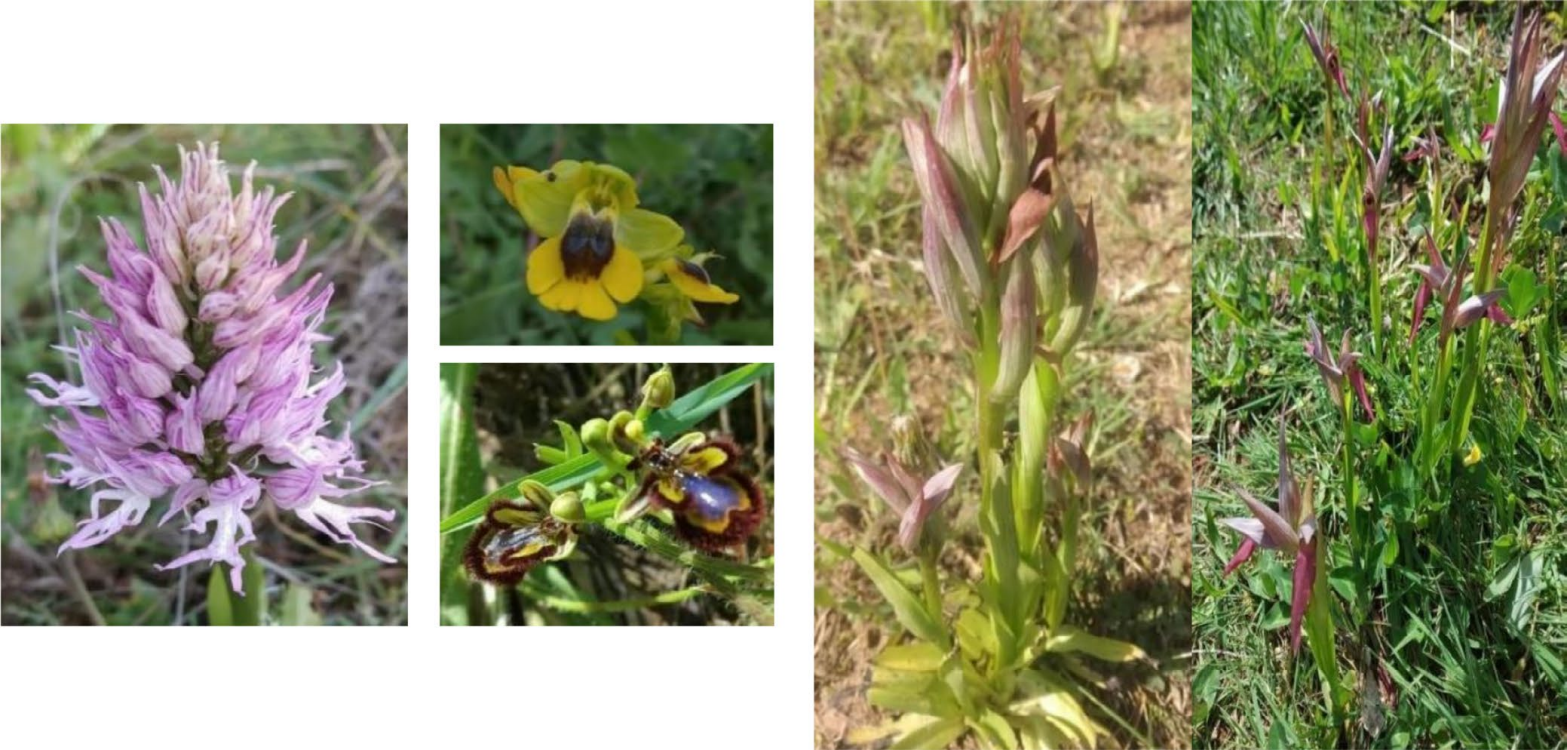
Orchidaceae species observed in the study area. From left to right: *Ochis italica* Poir, *Ophrys lutea* Cav (above), *Ophrys* *speculum*. Link (below), *Serapias parviflora* Parl. and *Serapias lingua* L. All photographs were taken by the authors.

Two species, *Cyperus eragrostis* Lam. and *Valerianella microcarpa* Loisel., which were highlighted by López‐Tirado (2018) as species to be sought out in this municipality as they had been cited previously from some adjacent municipalities. *C. eragrostis* , native to America, whose presence in the province of Córdoba was outside the municipal limits of the city (Devesa 2015). Additionally, it is noteworthy that this park has presence of five orchid species during the studied period: *Orchis italica* Poir., *Ophrys speculum* Link., *Ophrys lutea* Cav., *Serapias parviflora* Parl. and *Serapias lingua* L. (Figure 2). Vázquez (2009) supports that the study of the taxa of this family, their conservation and stability in landscapes, is essential for the resilience of vegetation and landscape. This is particularly true for the Orchidaceae family, which is one of the most damaged due to the destruction of their habitat, their collection, and the fact that their development depends on biological relationships. They need a balanced ecosystem (Faria 2018), as they are bioindicators in the conservation of habitats.

### 3.4 Similarity in the Floristic Composition of the Most Managed Areas

**FIGURE 3:**
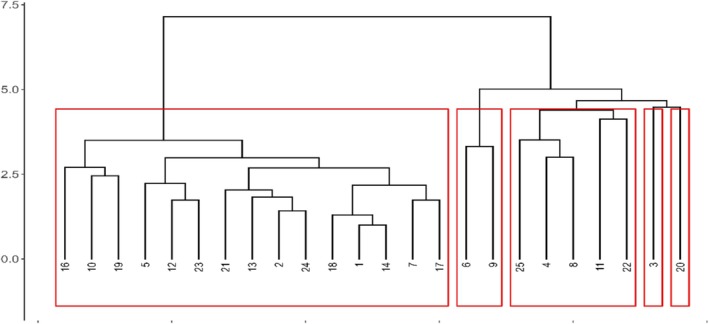
Dendogram of the similarity between random points.

Most of the surface of this study area is covered by grassland, which is subject to intensive management. This has a significant impact on the biodiversity and floristic composition of the area. Analysis of the grassland is therefore fundamental to understand how management practices affect biodiversity and ecosystem services. A total of 64 species were recorded in the presence sampling of the 25 randomly selected points. *Cynodon dactylon* L. was present in 84% of the sampled points, *Paspalum dilatatum* Poir. in 56%, *Plantago lanceolata* L. in 52%, *Trifolium repens* L. in 52%, and *Taraxacum leucopodum* G.E.Haglund. in 32%. The Jaccard method (Figure 3) revealed the presence of two major groupings, which in turn were found to be subdivided. This suggests that the species composition between the two groups is comparable, while the subgroups within each group exhibit distinct species compositions.

Dendrogram showing the similarity among the 25 randomly selected sampling points based on presence data of recorded species. Similarity was calculated using the Jaccard index. The horizontal axis represents the level of dissimilarity among samples. Red blocks indicate the five clusters identified according to the selected similarity threshold (see Table 6), grouping sampling points with comparable floristic composition.

**TABLE 6:** Species composition of each cluster.

Cluster	Points	Species
1	[1, 2, 5, 7, 10, 12–14, 16–19, 21, 23, 24]	*Trifolium repens* , *Cynodon dactylon* , *Paspalum dilatatum* , *Plantago lanceolata* , *Taraxacum leucopodum*, *Medicago sativa* , *Plantago lagopus*, *Medicago polymorpha* , *Convolvulus arvensis* , *Scorpiurus muricatus* , *Prunella vulgaris* , *Torilis arvensis* subsp. *neglecta*, *Erigeron bonariensis* ., *Lotus glaber* , *Mentha suaveolens* , *Daucus carota* , *Torilis nodosa* , *Urospermum picroides* , *Erigeron bonariensis* , *Washingtonia robusta* , *Anagallis monelli*
2	[6, 9]	*Cynodon dactylon* , *Plantago lagopus*, *Medicago polymorpha* , *Leontodon longirostris*, *Scorpiurus muricatus* , *Crepis vesicaria* subsp. *taraxacifolia*, *Rostraria cristata* , *Trifolium scabrum* , *Astragalus hamosus* , *Pulicaria arabica* subsp. *hispanica*, *Hirschfeldia incana* , *Trifolium tomentosum* , *Medicago doliata* , *Filago pyramidata* , *Malva parviflora* , *Trifolium resupinatum* , *Verbascum sinuatum* , *Trifolium glomeratum*
3	[4, 8, 11, 22, 25]	*Trifolium repens* , *Cynodon dactylon* , *Plantago lanceolata* , *Taraxacum leucopodum*, *Medicago sativa* , *Plantago lagopus, Leontodon longirostris*, *Catapodium rigidum* subsp. *rigidum*, *Scorpiurus muricatus* , *Crepis vesicaria* subsp. *taraxacifolia*, *Rostraria cristata* , *Prunella vulgaris* , *Dactylis glomerata* subsp. *hispanica*, *Trifolium scabrum* , *Astragalus hamosus* , *Crepis vesicaria* , *Crepis capillaris* , *Avena barbata* , *Pulicaria arabica* subsp. *hispanica*, *Medicago doliata* , *Filago pyramidata* , *Brachypodium distachyon* , *Linum tenue, Paronychia argentea*, *Chamaesyce maculata* , *Foeniculum vulgare* , *Eryngium campestre* , *Carlina racemosa*, *Polycarpon tetraphyllum* subsp. *tetraphyllum*, *Convolvulus althaeoides* , *Euphorbia falcata* subsp. *falcata, Helianthemum ledifolium*, *Anagallis arvensis* , *Vulpia myuros*
4	[3]	*Medicago sativa* , *Convolvulus arvensis* , *Dactylis glomerata* subsp. *hispanica*, *Torilis arvensis* , *Crepis vesicaria* subsp. *taraxacifolia*, *Avena sterilis* , *Medicago orbicularis* , *Cichorium intybus* , *Lactuca serriola*
5	[20]	*Cynodon dactylon* , *Leontodon longirostris*, *Crepis vesicaria* subsp. *taraxacifolia*, *Dactylis glomerata* subsp. *hispanica*, *Avena barbata* , *Linum tenue, Andryala integrifolia, Sanguisorba verrucosa, Hedypnois rhagadioloides*, *Bromus hordeaceus* , *Bromus matritensis* , *Daucus crinitus*, *Allium paniculatum*

### 3.5 Health Implications

**FIGURE 4:**
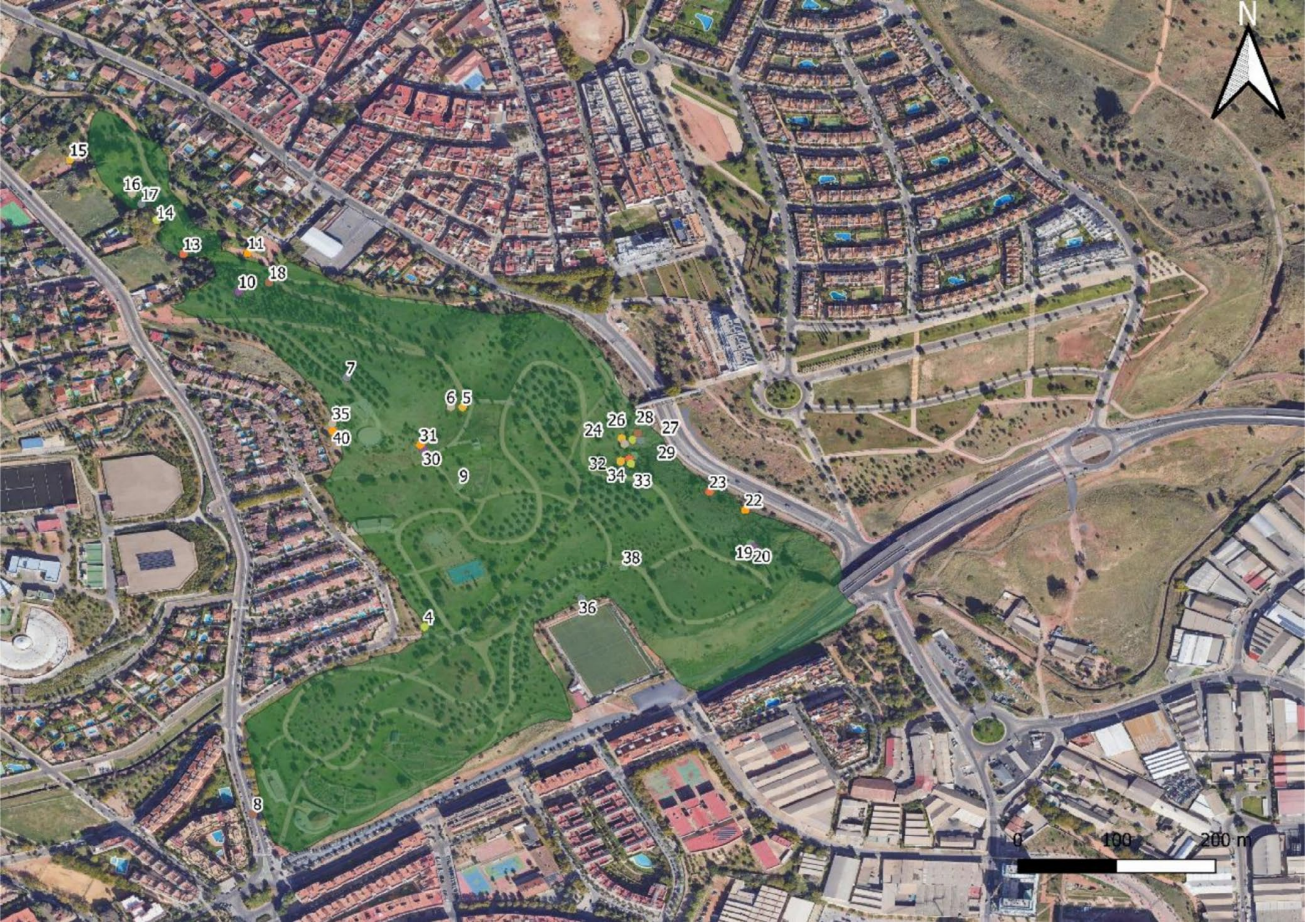
Inventories showing the occurrence of more represented species of the family Asteraceae and Poaceae. The full floristic inventory is available in Appendix S1.

In our floristic inventory, two families, Poaceae and Asteraceae stood out for both species richness and individual occurrence, with 28 and 37 species, respectively. Poaceae are known for their allergenic potential and aerobiological relevance due to their anemophilous pollination and high pollen production, are relevant in urban aerobiology (Kupias et al. 1981). Among Poaceae, species such as *Dactylis glomerata* subsp. *hispanica* (Roth) Nyman, *Piptatherum miliaceum* (L.) Coss, and *Vulpia geniculata* (L.) Link were detected in managed areas, all of which are documented as producing the high concentration of pollen grains (Prieto‐Baena et al. 2003; Romero‐Morte et al. 2018). In contrast, allergenicity within Asteraceae is mainly associated with anemophilous taxa, that is, *Artemisia* L. and *Ambrosia* L. (Fernández et al. 1993; Ziska et al. 2003); in our study area *Taraxacum leucopodum* G.E.Haglund and *Andryala integrifolia* L., were notably frequent in the samples (Figure 4), but are predominantly entomophilous and are generally considered to have low aerobiological impact (Magyar 2023; Preda et al. 2024).

Additionally, two spontaneous species of the Oleaceae family, *Fraxinus angustifolia* Vahl. and *Olea europaea* var. *sylvestris* (Mill.) Lehr., were recorded. These taxa are known to contribute allergenic pollen during overlapping flowering periods (Cebrino et al. 2017), with *Fraxinus* sp. flowering earlier than *Olea* sp., potentially extending the allergy season. Although our study did not measure atmospheric pollen concentrations directly, the local presence of these taxa provides relevant baseline information for future environmental health monitoring and allergen mitigation strategies in urban green spaces.

Pollen from the Amaranthaceae family has been identified as potentially allergenic (Elvira‐Rendueles et al. 2017). In the present study, the most represented species were *Amaranthus blitoides* S. Watson and *Amaranthus viridis* L., both of which have documented allergenic potential and evidence of cross‐reactivity with other types of pollen (Galán et al. 1989; Cariñanos et al. 2014; Villalba et al. 2014). These findings suggest that even spontaneous vegetation in urban parks can include species with implications for respiratory health, reinforcing the importance of integrating allergenicity into green space management planning.

## 4 Conclusion

This study provides a detailed floristic inventory of the spontaneous flora of “La Asomadilla” urban park in Córdoba, highlighting a high level of floristic diversity and the presence of species of ecological and health interest. Most of the species identified are therophytes, well adapted to the arid conditions of the Mediterranean climate. The discovery of species never cited before for the municipality and the presence of orchids underscore the ecological value of this space. Importantly, this research emphasizes the novelty and significance of studying spontaneous wild flora in an urban park, an aspect usually overlooked in favor of ornamental or tree species. These results reveal the essential role of this accompanying flora, particularly herbaceous vegetation, in mitigating urban heat, enhancing CO_2_ capture, and improvising biodiversity, making it preferable to maintain bare soils under trees and shrubs.

Nevertheless, the study presents some limitations. Species compositions may change over time due to garden management practices, and the presence of spontaneous flora can vary between different types of urban green spaces, limiting the extrapolation of results. Future comparative studies across diverse gardens and urban areas would therefore be valuable to understand how wild flora diversity responds to different contexts.

Finally, the choice of Córdoba as a study site reinforces the relevance of this research, as the city increasingly faces long and intense heatwaves. In this context, conserving and promoting spontaneous flora in urban parks emerges as a key strategy for biodiversity conservation, climate change mitigation, and public health improvement.

## Funding

The authors have no relevant financial or non‐financial intereststo disclose.

## Conflicts of Interest

The authors declare no conflicts of interest.

## Supporting information


**Table S1:** Flora species and their corresponding ecological characteristics. The species names have been verified according to the World Flora Online Consortium.
**Appendix S1:** Complete list of plant species recorded in the study area.

## Data Availability

Data are available from the Zenodo Digital Repository: https://zenodo.org/records/18961238.
