# Supplementary material for: Analysis of Spontaneous Plant Species in an Urban Green Space in Southern Spain
Source: Plant Environ Interact. 2026 Apr 30;7(3):e70144. doi: 10.1002/pei3.70144 (PMC13130033; doi:10.1002/pei3.70144)
Supplement: Supplementary file 1 — Table S1: Flora species and their corresponding ecological characteristics. The species names have been verified according to the World Flora Online Consortium. Appendix S1: Complete list of plant species recorded in the study area. [file PEI3-7-e70144-s001.docx]

**Supplementary “Information for Floristic Catalogue of spontaneous species in an urban green space in Southern Spain”**

**Table S1.** Flora species and their corresponding ecological characteristics. The species names have been verified according to the World Flora Online Consortium.

| Scientific name | Familiy Name | Life form | Phyto-geographical origin |
| --- | --- | --- | --- |
| *Acanthus mollis* L*.* | Acanthaceae | He | Med |
| *Aegilops neglecta* Req. ex Bertol. | Poaceae | Th | Ir.-Tur |
| *Ajuga iva*(L.) Schreb. | Lamiaceae | Ch | Euro.-Circum.-Med |
| *Allium paniculatum* L. | Amaryllidaceae | Ge | Euro.-Circum.-Med |
| *Amaranthus blitoides*S.Watson | Amaranthaceae | Th | Holar |
| *Amaranthus viridis*L. | Amaranthaceae | Th | Neotrop |
| *Anagallis arvensis*L. | Primulaceae | Th | Med |
| *Anagallis monelli*L. | Primulaceae | Th | Euro.-Circum.-Med |
| *Anagyris foetida*L. | Fabaceae | Ph | Med |
| *Andryala integrifolia*L. | Asteraceae | Th | Med |
| *Anthemis arvensis*L. | Asteraceae | Th | Med |
| *Antirrhinum barrelieri* Boreau. | Plantaginaceae | Th | Med |
| *Apium nodiflorum*(L.) Rchb.fil. | Apiaceae | Hy | Later.-Circum.-Med |
| *Arisarum simorrhinum*Durieu. | Araceae | Ge | Med |
| *Aristolochia baetica*L. | Aristolochiaceae | Ph | Iber.-Magre |
| *Arum italicum*Mill. | Araceae | Ge | Euro.-Circum.-Med |
| *Arundo donax*L. | Poaceae | Hy | Cosm |
| *Asparagus acutifolius*L. | Asparagaceae | Ge | Euro.-Circum.-Med |
| *Asparagus albus*L. | Asparagaceae | Ph | Euro.-Circum.-Med |
| *Asperula hirsuta*Desf. | Rubiaceae | He | Iber.-Afr |
| *Asphodelus fistulosus*L. | Asphodelaceae | He | Paleotrop |
| *Astragalus hamosus*L. | Fabaceae | Th | Ir.-Tur |
| *Atractylis cancellata*L. | Asteraceae | Th | Euro.-Circum.-Med |
| *Avena barbata*Pott ex Link. | Poaceae | Th | Ir.-Tur |
| *Avena sterilis* L. | Poaceae | Th | Ir.-Tur |
| *Bartsia trixago* L. | Orobanchaceae | Th | Later.-Circum.-Med |
| *Bellis sylvestris*Cirillo*.* | Asteraceae | He | Med |
| *Blackstonia perfoliata* (L.) Huds. | Gentianaceae | Th | Med |
| *Brachypodium distachyon*(L.) P.Beauv. | Poaceae | Th | Ir.-Tur |
| *Bromus diandrus*Roth*.* | Poaceae | Th | Later.-Circum.-Med |
| *Bromus hordeaceus* L*.* | Poaceae | Th | Cosm |
| *Bromus madritensis* L. | Poaceae | Th | Eurosib.-Med.-Ir.-Tur |
| *Calendula arvensis* L*.*subsp*. arvensis* | Asteraceae | Th | Later.-Circum.-Med |
| *Capsella bursa-pastoris* L. | Brassicaceae | Th | Med.-Ir.-Tur |
| *Cardamine hirsuta*L. | Brassicaceae | Th | Cosm |
| *Carduus pycnocephalus* L. | Asteraceae | He | Ir.-Tur |
| *Carex divisa*Huds*.* | Cyperaceae | Ge | Sub.-Med |
|  |  |  |  |
| **Table S1.** (continued) |  |  |  |
| Scientific name | Familiy Name | Life form | Phyto-geographical origin |
| *Carlina corymbosa*L. subsp*. hispanica*(Lam.) O. Bolòs & Vigo | Asteraceae | Th | Med |
| *Carlina gummifera*Less*.* | Asteraceae | Th | Med |
| *Carlina racemosa* L. | Asteraceae | Th | Med |
| [*Catapodium rigidum* (L.) C.E. Hubb. in J.G. Dony subsp*. rigidum*](https://www.biodiversidadcanarias.es/biota/especie/F01923) | Poaceae | Th | Later.-Circum.-Med |
| *Celtis australis*L. | Cannabaceae | Ph | Med |
| *Centaurea pullata* L. | Asteraceae | Th | Iber.-Afr |
| *Centaurium grandiflorum* subsp*. majus* (Hoffmanns. & Link) Z.Díaz | Gentianaceae | He | Med |
| *Cerastium glomeratum* Thuill. | Caryophyllaceae | Th | Later.-Circum.-Med |
| *Ceterach officinarum* subsp*. officinarum* Willd., 1804 | Aspleniaceae | He | Cosm |
| *Chamaemelum fuscatum* (Gren. & Godr.) Vasc. | Asteraceae | Th | Med |
| *Chamaesyce canescens (*L.) Prokh. subsp*. canescens* | Euphorbiaceae | Th | Later.-Circum.-Med |
| *Chamaesyce maculata* (L.) Small | Euphorbiaceae | Th | Holar |
| *Cichorium intybus*L. | Asteraceae | Th | Cosm |
| *Clematis flammula* L. | Ranunculaceae | Ph | Later.-Circum.-Med |
| *Convolvulus althaeoides* L. | Convolvulaceae | He | Euro.-Circum.-Med |
| *Convolvulus arvensis* L. | Convolvulaceae | He | Cosm |
| *Coronopus didymus*(L.) Sm. | Brassicaceae | Th | Holar |
| *Crataegus monogyna*Jacq. | Rosaceae | Ph | Iber.-Afr |
| *Crepis capillaris*(L.) Wallr. | Asteraceae | Th | Sub.-Med |
| *Crepis vesicaria* subsp*. taraxacifolia* (Thuill.) Thell. | Asteraceae | Th | Sub.-Med |
| *Cynara humilis*L. | Asteraceae | He | Iber.-Afr |
| *Cynodon dactylon*(L.) Pers. | Poaceae | He | Cosm |
| *Cynoglossum creticum*Mill. | Boraginaceae | He | Later.-Circum.-Med |
| *Cyperus eragrostis*Lam. | Cyperaceae | He | Holar |
| *Cyperus longus* L. | Cyperaceae | He | Holar |
| *Dactylis glomerata* subsp*. hispanica* (Roth) Nyman | Poaceae | He | Euro.-Circum.-Med |
| *Daphne gnidium*L. | Thymelaeaceae | Ph | Euro.-Circum.-Med |
| *Daucus carota*L. | Apiaceae | He | Cosm |
| *Daucus crinitus*Desf*.* | Apiaceae | He | Cosm |
| *Dichondra micrantha*Urb. | Convolvulaceae | Ge | Paleotrop |
| *Digitaria sanguinalis*(L.) Scop. | Poaceae | Th | Cosm |
| *Diplotaxis catholica* (L.) DC. | Brassicaceae | Th | Iber.-Afr |
| *Drimia maritima* (L.) Stearn | Asparagaceae | Ge | Med |
| *Echium plantagineum* L. | Boraginaceae | Th | Later.-Circum.-Med |
| *Eleusine indica*(L.) Gaertn. | Poaceae | Th | Cosm |
| *Erigeron bonariensis* L. | Asteraceae | Th | Holar |
| *Erigeron canadensis*L. | Asteraceae | Th | Holar |
| *Erigeron sumatrensis* Retz. | Asteraceae | Th | Holar |
| *Erodium cicutarium* (L.) L'Hér. | Geraniaceae | Th | Later.-Circum.-Med |
| **Table S1.** (continued) |  |  |  |
| Scientific name | Familiy Name | Life form | Phyto-geographical origin |
| *Erodium malacoides* subsp*. malacoides* | Geraniaceae | Th | Later.-Circum.-Med |
| *Erodium moschatum* (L.) L'Hér. | Geraniaceae | Th | Later.-Circum.-Med |
| *Eryngium campestre* L. | Apiaceae | He | Later.-Circum.-Med |
| *Euphorbia exigua*L*.* subsp*. exigua* | Euphorbiaceae | Th | Med |
| *Euphorbia falcata*L. subsp*. falcata* | Euphorbiaceae | Th | Med |
| *Euphorbia helioscopia* L. subsp*. helioscopia* | Euphorbiaceae | Th | Cosm |
| *Euphorbia sulcata* Lens ex Loisel. | Euphorbiaceae | Th | Med |
| *Ferula communis* subsp*. catalaunica* (Pau) Sánchez-Cux. & M.Bernal | Apiaceae | He | Neotrop |
| *Festuca ampla*Hack. | Poaceae | He | Med |
| *Ficus carica*L. | Moraceae | Ph | Ir.-Tur |
| *Filago pyramidata* L. | Asteraceae | Th | Later.-Circum.-Med |
| *Foeniculum vulgare*Mill. | Apiaceae | He | Holar |
| *Fraxinus angustifolia*Vahl | Oleaceae | Ph | Cosm |
| *Fumaria capreolata*L*.* | Papaveraceae | Th | Later.-Circum.-Med |
| *Fumaria officinalis* L. | Papaveraceae | Th | Paleotrop |
| *Galactites tomentosus*Moench | Asteraceae | Th | Euro.-Circum.-Med |
| *Galium aparine*L. | Rubiaceae | Th | Euras |
| *Galium murale*(L.) All. | Rubiaceae | Th | Euro.-Circum.-Med |
| *Galium parisiense* subsp*. parisiense* L., 1753 | Rubiaceae | Th | Later.-Circum.-Med |
| *Galium verrucosum* var*. verrucosum* Huds., 1767 | Rubiaceae | Th | Med |
| *Gaudinia fragilis* (L.) P.Beauv. | Poaceae | Th | Med |
| *Geranium dissectum*L. | Geraniaceae | Th | Med |
| *Geranium molle*L. | Geraniaceae | Th | Cosm |
| *Gladiolus italicus*Mill. | Iridaceae | Ge | Med |
| *Glebionis coronaria*(L.) Tzvelev | Asteraceae | Th | Euro.-Circum.-Med |
| *Glossopappus macrotus* (Durand) Briq. | Asteraceae | Ph | Iber.-Afr |
| *Gynandriris sisyrinchium*(L.) Parl. | Iridaceae | Ge | Euro.-Circum.-Med |
| *Hedypnois rhagadioloides* (L.) F.W.Schmidt | Asteraceae | Th | Euro.-Circum.-Med |
| *Helianthemum ledifolium*(L.) Mill. | Cistaceae | Th | Euro.-Circum.-Med |
| *Heliotropium europaeum*L. | Heliotropiaceae | Th | Ir.-Tur |
| *Hirschfeldia incana*(L.) Lagr.-Foss. | Brassicaceae | He | Later.-Circum.-Med |
| *Hypericum perforatum*L. | Hypericaceae | He | Paleotrop |
| *Hyparrhenia sinaica* (Delile) Llauradó ex G.López | Poaceae | He | Med.- Paleotrop |
| *Lactuca serriola*L. | Asteraceae | He | Med |
| *Lagoecia cuminoides*L. | Apiaceae | Th | Med |
| *Lamarckia aurea* (L.) Moench | Poaceae | Th | Ir.-Tur |
| *Lamium amplexicaule*L. | Lamiaceae | Th | Holar |
| *Lathyrus cicera* L. | Fabaceae | Th | Med |
| *Laurus nobilis* L. | Lauraceae | Ph | Med |
| *Lavatera trimestris* L. | Malvaceae | Th | Med |
| *Leontodon longirostris* (Finch & P.D.Sell) Talavera | Asteraceae | Th | Euras |
| **Table S1.** (continued) |  |  |  |
| Scientific name | Familiy Name | Life form | Phyto-geographical origin |
| *Linum tenue*Desf. | Linaceae | Th | Med |
| *Lonicera japonica*Thunb. | Caprifoliaceae | Ph | Holar |
| *Lotus glaber*Mill. | Fabaceae | He | Paleotrop |
| *Malva hispanica*L. | Malvaceae | Th | Med |
| *Malva nicaeensis*All*.* | Malvaceae | He/Th | Euro.-Circum.-Med |
| *Malva parviflora*L. | Malvaceae | Th | Euro.-Circum.-Med |
| *Medicago arabica* (L.) Huds. | Fabaceae | Th | Holar |
| *Medicago doliata*Carmign*.* | Fabaceae | Th | Euro.-Circum.-Med |
| *Medicago minima*(L.) Bartal. | Fabaceae | Th | Ir.-Tur |
| *Medicago orbicularis* (L.) Bartal | Fabaceae | Th | Med |
| *Medicago polymorpha*L. | Fabaceae | Th | Med |
| *Medicago sativa* L. | Fabaceae | Th | Ir.-Tur |
| *Medicago truncatula*Gaertn. | Fabaceae | Th | Euro.-Circum.-Med |
| *Melia azedarach*L. | Meliaceae | Ph | Holar |
| *Melica ciliata* subsp*. magnolii* (Godr. & Gren.) K.Richt. | Poaceae | He | Ir.-Tur |
| *Melilotus indicus*(L.) All. | Fabaceae | Th | Later.-Circum.-Med |
| *Mentha suaveolens*Ehrh*.* | Lamiaceae | He | Later.-Circum.-Med |
| *Mercurialis ambigua*L.f. | Euphorbiaceae | Th | Holar |
| *Micromeria graeca* (L.) Benth. ex Rchb. | Lamiaceae | Ch | Euro.-Circum.-Med |
| *Morus alba* L. | Moraceae | Ph | Ir.-Tur |
| *Muscari neglectum* Guss. ex Ten. | Asparagaceae | Ge | Med |
| *Myrtus communis* L. | Myrtaceae | Ph | Med |
| *Oenothera rosea*Aiton | Onagraceae | He | Holar |
| *Olea europaea var. sylvestris* (Mill.) Lehr | Oleaceae | Ph | Med |
| *Onobrychis viciifolia* Scop. | Fabaceae | Ch | Med |
| *Ophrys lutea*Cav. | Orchidaceae | Ge | Med |
| *Ophrys speculum*Link | Orchidaceae | Ge | Med |
| *Orchis italica*Poir*.* | Orchidaceae | Ge | Med |
| *Ornithogalum narbonense* L. | Asparagaceae | Ge | Med |
| *Orobanche nana* (Reut.) Beck | Orobanchaceae | Th-par. | Cosm |
| *Osyris alba*L*.* | Santalaceae | Ph | Later.-Circum.-Med |
| *Oxalis pes-caprae*L. | Oxalidaceae | Ge | Cosm |
| *Pallenis spinosa* (L.) Cass. | Asteraceae | He | Later.-Circum.-Med |
| *Papaver rhoeas*L. | Papaveraceae | Th | Later.-Circum.-Med |
| *Parentucellia latifolia* (L.) Caruel | Orobanchaceae | Th | Med |
| *Parietaria judaica* L. | Urticaceae | Th | Later.-Circum.-Med |
| *Paronychia argentea*Lam. | Caryophyllaceae | Ch | Med |
| *Paspalum dilatatum* Poir. | Poaceae | He | Cosm |
| *Phagnalon saxatile* (L.) Cass. | Asteraceae | Ch | Med |
| *Phlomis lychnitis*L. | Lamiaceae | Ch | Med |
| *Phlomis purpurea*L. | Lamiaceae | Ch | Iber.-Afr |
| *Phoenix canariensis* H.Wildpret | Arecaceae | Ph | Paleotrop |
| **Table S1.** (continued) |  |  |  |
| Scientific name | Familiy Name | Life form | Phyto-geographical origin |
| *Pinus pinea*L. | Pinaceae | Ph | Med |
| *Piptatherum miliaceum* (L.) Coss. | Poaceae | Th | Ir.-Tur |
| *Pistacia lentiscus* L*.* | Anacardiaceae | Ph | Holar |
| *Plantago coronopus*L. | Plantaginaceae | He/Th | Later.-Circum.-Med |
| *Plantago lagopus* L. | Plantaginaceae | Th | Euro.-Circum.-Med |
| *Plantago lanceolata* L. | Plantaginaceae | He | Holar |
| *Plantago major*L. | Plantaginaceae | He | Cosm |
| *Plantago serraria* L. | Plantaginaceae | He | Cosm |
| *Poa annua*L. | Poaceae | Th | Cosm |
| *Poa bulbosa* L. | Poaceae | He | Cosm |
| *Poa infirma*Kunth | Poaceae | Th | Cosm |
| *Polycarpon tetraphyllum* (L.) L. subsp*. tetraphyllum* | Caryophyllaceae | Th | Later.-Circum.-Med |
| *Polygonum aviculare*L. | Polygonaceae | Th | Cosm |
| *Portulaca oleracea*L. | Portulacaceae | Th | Cosm |
| *Prospero autumnale*(L.) Speta | Asparagaceae | Ge | Med |
| *Prunella vulgaris* L. | Lamiaceae | He | Holar |
| *Pulicaria arabica* subsp*. hispanica* (Boiss.) Murb. | Asteraceae | Th | Med |
| *Quercus ilex* var*. ballota* (Desf.) Bonafé | Fagaceae | Ph | Med |
| *Ranunculus bullatus*L. | Ranunculaceae | Th | Cosm |
| *Ranunculus ficaria* L. subsp*. ficaria* | Ranunculaceae | Th | Later.-Circum.-Med |
| *Ranunculus muricatus*L. | Ranunculaceae | Th | Later.-Circum.-Med |
| *Raphanus raphanistrum* subsp*. raphanistrum* | Brassicaceae | Th | Later.-Circum.-Med |
| *Reichardia intermedia* (Jan ex DC.) Cout. | Asteraceae | Th | Euro.-Circum.-Med |
| *Rhamnus alaternus*L. | Rhamnaceae | Ph | Med |
| *Romulea ramiflora* Ten subsp*. ramiflora* | Iridaceae | Ge | Med |
| *Rostraria cristata* (L.) Tzvelev | Poaceae | Th | Cosm |
| *Rubus ulmifolius*Schott | Rosaceae | Ph | Later.-Circum.-Med |
| *Rumex conglomeratus*Murray | Polygonaceae | He | Cosm |
| *Rumex crispus* L. | Polygonaceae | He | Cosm |
| *Rumex induratus*Boiss. & Reut. | Polygonaceae | He | Med |
| *Rumex pulcher*L*.* subsp*. pulcher* | Polygonaceae | He | Later.-Circum.-Med |
| *Sagina apetala*Ard*.* | Caryophyllaceae | Th | Later.-Circum.-Med |
| *Salvia verbenaca*L. | Lamiaceae | He | Sub.-Med |
| *Sanguisorba verrucosa*(G.Don) Ces. | Rosaceae | He | Euro.-Circum.-Med |
| *Scabiosa atropurpurea*L*.* | Caprifoliaceae | He | Euro.-Circum.-Med |
| *Scirpoides holoschoenus* (L.) Soják | Cyperaceae | Ge | Later.-Circum.-Med |
| *Scorpiurus muricatus*L. | Fabaceae | Th | Med |
| *Sedum caespitosum* (Cav.) DC. | Crassulaceae | Ch | Euro.-Circum.-Med |
| *Sedum sediforme*(Jacq.) Pau | Crassulaceae | Th | Med |
| *Selaginella denticulata* (L.) Spring | Selaginellaceae | Ch | Med |
| *Senecio vulgaris*L. | Asteraceae | Th | Later.-Circum.-Med |
| *Serapias lingua*L. | Orchidaceae | Ge | Med |
| **Table S1.** (continued) |  |  |  |
| Scientific name | Familiy Name | Life form | Phyto-geographical origin |
| *Serapias parviflora*Parl*.* | Orchidaceae | Ge | Med |
| *Setaria pumila* (Poir.) Roem. & Schult. | Poaceae | Th | Cosm |
| *Sherardia arvensis*L. | Rubiaceae | Th | Later.-Circum.-Med |
| *Silene colorata*Poir*.* | Caryophyllaceae | Th | Later.-Circum.-Med |
| *Silene gallica* L. | Caryophyllaceae | Th | Med |
| *Silybum marianum*(L.) Gaertn. | Asteraceae | He | Ir.-Tur |
| *Sinapis alba* subsp*. mairei* (H.Lindb.) Maire | Brassicaceae | Th | Euro.-Circum.-Med |
| *Sisymbrium officinale* (L.) Scop. | Brassicaceae | Th | Holar |
| *Smyrnium olusatrum* L*.* | Apiaceae | Th | Sub.-Med |
| *Sonchus asper* subsp*. asper* (L.) Hill | Asteraceae | Th | Cosm |
| *Sonchus oleraceus*L. | Asteraceae | Th | Cosm |
| *Sorghum halepense*(L.) Pers. | Poaceae | Ge | Med |
| *Spergularia purpurea* (Pers.) G.Don | Caryophyllaceae | Th | Med |
| *Stachys arvensis*(L.) L. | Lamiaceae | Th | Med |
| *Stachys ocymastrum* (L.) Briq. | Lamiaceae | Th | Med |
| *Stellaria media*(L.) Vill. | Caryophyllaceae | Th | Cosm |
| *Stipa capensis*Thunb*.* | Poaceae | Th | Euro.-Circum.-Med |
| *Symphyotrichum squamatum* (Spreng.) G.L.Nesom | Asteraceae | He | Holar |
| *Taraxacum leucopodum* G.E.Haglund | Asteraceae | He | Holar |
| *Torilis arvensis* subsp*. neglecta* (Spreng.) Thell. | Apiaceae | Th | Later.-Circum.-Med |
| *Torilis nodosa* (L.) Gaertn. | Apiaceae | Th | Ir.-Tur |
| *Trachelium caeruleum* L. subsp. *caeruleum* | Campanulaceae | Ch | Med |
| *Tragopogon angustifolius* Bellardi ex Willd. | Asteraceae | Th | Later.-Circum.-Med |
| *Trifolium angustifolium* L. | Fabaceae | Th | Later.-Circum.-Med |
| *Trifolium campestre* Schreb*.* | Fabaceae | Th | Holar |
| *Trifolium glomeratum*L. | Fabaceae | Th | Later.-Circum.-Med |
| *Trifolium pratense* L*.* subsp*. pratense* | Fabaceae | Th | Euro.-Sib |
| *Trifolium repens*L. | Fabaceae | He | Holar |
| *Trifolium resupinatum* L. | Fabaceae | Th | Later.-Circum.-Med |
| *Trifolium scabrum*L. | Fabaceae | Th | Sub.-Med |
| *Trifolium stellatum*L. | Fabaceae | Th | Later.-Circum.-Med |
| *Trifolium tomentosum* L*.* | Fabaceae | Th | Euro.-Circum.-Med |
| *Tripodion tetraphyllum*(L.) Fourr. | Fabaceae | Th | Med |
| *Ulmus pumila*L. | Ulmaceae | Ph | Euras |
| *Umbilicus rupestris* (Salisb.) Dandy | Crassulaceae | Ge | Sub.-Med |
| *Urospermum picroides* (L.) Scop. ex F.W.Schmidt | Asteraceae | Th | Med |
| *Urtica urens L.* | Urticaceae | Th | Med |
| *Valerianella microcarpa* Loisel*.* | Caprifoliaceae | Th | Euro.-Circum.-Med |
| *Verbascum sinuatum* L. | Scrophulariaceae | He | Later.-Circum.-Med |
| *Verbena officinalis* L. | Verbenaceae | He | Holar |
| *Veronica arvensis*L. | Plantaginaceae | Th | Cosm |
| *Veronica cymbalaria*Bodard | Plantaginaceae | Th | Later.-Circum.-Med |
| **Table S1.** (continued) |  |  |  |
| Scientific name | Familiy Name | Life form | Phyto-geographical origin |
| *Veronica persica*Poir*.* | Plantaginaceae | Th | Cosm |
| *Veronica polita*Fr. | Plantaginaceae | Th | Ir.-Tur |
| *Vicia faba*L. | Fabaceae | Th | Med |
| *Vicia sativa*L. subsp*. sativa* | Fabaceae | Th | Med.-Euras |
| *Vinca difformis* Pourr subsp*. difformis* | Apocynaceae | Ch | Med |
| *Vulpia myuros*(L.) C.C.Gmel. | Poaceae | Th | Cosm |
| *Washingtonia robusta*H.Wendl*.* | Arecaceae | Ph | Holar |

Life form. *He* Hemicryptophytes; *Th* Therophyte; *Ch* Chamaephytes; *Ge* Geophytes; *Ph* Phanerophyte; *Hy* Hydrophytes. Phyto-geographical. *Med* Mediterranean; *Later.-Circum.-Med:* Later.-Circum.-Mediterranean; *Cosm* Cosmopolitan; *Euro.-Circum.-Med* Euro.-Circum.-Mediterranean; *Holar* Holarctic; *Ir.-Tur* Irano-Turanian; *Iber.-Afr* Ibero-African; *Sub.-Med* Sub Mediterranean; *Paleotrop* Paleotropical; *Euras* Eurasian; *Neotrop* Neotropical*; Euro.-Sib* Euro-Siberian; *Eurosib.-Med.-Ir.-Tur* Euro-Siberian-Mediterranean-Irano-Tauranien; *Iber.-Magre* Ibero-Maghrebi; *Med.-Euras* Mediterranean-Eurasian; *Med.-Ir.-Tur* Mediterranean-Irano-Turanian; *Med.-Paleotrop* Mediterranean- Paleotropical.

**Supplementary Appendix S1. Complete list of plant species recorded in the study area.**

Inventory 4.

- *Crepis vesicaria* subsp*. taraxacifolia*
- *Centaurea pullata*
- *Anthemis arvensis*
- *Vulpia myuros*

Inventory 5.

- *Poa annua*
- *Taraxacum leucopodum*

Inventory 6.

- *Leontodon longirostris*

Inventory 7.

- *Taraxacum leucopodum*
- *Chamaemelum fuscatum*
- *Crepis vesicaria* subsp*. taraxacifolia*

Inventory 8.

- *Taraxacum leucopodum*
- *Cynodon dactylon*

Inventory 9.

- *Andryala integrifolia*
- *Crepis vesicaria* subsp*. taraxacifolia*
- *Calendula arvensis* subsp*. arvensis*
- *Hedypnois rhagadioloides*
- *Urospermun picroides*
- *Vulpia geniculata*

Inventory 10.

- *Crepis vesicaria* subsp*. taraxacifolia*
- *Pallenis spinosa*
- *Eryngium campestre*
- *Anthemis arvensis*
- *Filago pyramidata*
- *Centaurea pullata*
- *Dactylis glomerata* subsp*. hispanica*

Inventory 11.

- *Taraxacum leucopodum*
- *Poa annua*

Inventory 13.

- *Arundo donax*

Inventory 15.

- *Calendula arvensis* subsp*. arvensis*

Inventory 16.

- *Sonchus oleraceus*
- *Crepis vesicaria* subsp*. taraxacifolia*
- *Avena sterilis*

Inventory 17.

- *Sonchus oleraceus*
- *Eryngium campestre*
- *Crespis vesicaria* subsp*. taraxacifolia*
- *Avena barbata*
- *Avena sterilis*
- *Bromus diandrus*

Inventory 18.

- *Calendula arvensis* subsp*. arvensis*
- *Cynara humilis*
- *Crepis vesicaria* subsp*. taraxacifolia*
- *Leontodon longirostis*
- *Centaurea pullata*
- *Vulpia geniculata*

Inventory 19.

- *Carlina corymbosa* subsp*. hispanica*
- *Dactylis glomerata* subsp*. hispanica*
- *Avena sterilis*

Inventory 20.

- *Phagnalon saxatile*
- *Crepis vesicaria* subsp*. taraxacifolia*
- *Eryngium campestre*
- *Carlina corymbosa* subsp*. hispanica*
- *Andryala integrifolia*
- *Dactylis glomerata* subsp*. hispanica*
- *Avena sterilis*

Inventory 22.

- *Crepis vesicaria* subsp*. taraxacifolia*
- *Phagnalon saxatile*
- *Galactites tomentosus*
- *Andryala integrifolia*
- *Sonchus oleraceus*
- *Carlina corymbosa* subsp*. hispanica*
- *Eryngium campestre*
- *Hyparrhenia sinaica*

Inventory 23.

- *Crepis vesicaria* subsp*. taraxacifolia*
- *Leontodon longirostris*
- *Galactites tomentosus*
- *Andryala integrifolia*

Inventory 24.

- *Leontodon longirostris*
- *Atractylis cancellata*
- *Galactites tomentosus*
- *Brachypodium distachyon*

Inventory 25.

- *Galactites tomentosus*
- *Andryala integrifolia*
- *Tragopogon angustifolius*
- *Leontodon longirostris*
- *Vulpia myuros*

Inventory 26.

- *Andryala integrifolia*
- *Galactites tomentosus*
- *Erigeron sumatrensis*
- *Avena sterilis*

Inventory 27.

- *Pallenis spinosa*
- *Galactites tomentosus*
- *Atractylis cancellata*
- *Leontodon longirostris*
- *Phagnalon saxatile*
- *Hyparrhenia sinaica*

Inventory 28.

- *Phagnalon saxatile*
- *Galactites tomentosus*
- *Pallenis spinosa*
- *Reichardia intermedia*
- *Eryngium campestre*

Inventory 29.

- *Galactites tomentosus*
- *Crepis vesicaria* subsp*. taraxacifolia*
- *Erigeron canadensis*
- *Andryala integrifolia*
- *Dactylis glomerata* subsp*. hispanica*

Inventory 30.

- *Andryala integrifolia*
- *Eryngium campestre*
- *Atractylis cancellata*
- *Leontodon longirostis*
- *Reichardia intermedia*
- *Crepis vesicaria* susbp*. taraxacifolia*
- *Phagnalon saxatile*
- *Hyparrhenia sinaica*

Inventory 31.

- *Atractylis cancellata*
- *Leontodon longirostris*
- *Brachypodium distachyon*

Inventory 32.

- *Eryngium campestre*
- *Cynara humilis*
- *Pallenis spinosa*
- *Andryala integrifolia*
- *Dactylis glomerata* subsp*. hispanica*
- *Hordeum murinum* subsp. *leporinum*

Inventory 33.

- *Atractylis cancellata*
- *Cynara humilis*
- *Dactylis glomerata subsp. hispanica*

Inventory 34.

- *Pallenis spinosa*
- *Andryala integrifolia*
- *Cynara humilis*

Inventory 35.

- *Eryngium campestre*
- *Hordeum murinum* subsp*. leporinum*

Inventory 36.

- *Erigeron canadensis*
- *Sorghum halepense*

Inventory 37.

- *Andryala integrifolia*
- *Crepis vesicaria* subsp*. taraxacifolia*
- *Sorghum halepense*

Inventory 38.

- *Taraxacum leocopodum*
- *Crepis vesicaria* subsp*. taraxacifolia*
- *Leontodon longirostis*
- *Cynodon dactylon*

Inventory 39.

- *Carlina corymbosa* subsp*. hispanica*
- *Carlina gummifera*
- *Pallenis spinosa*
- *Galactites tomentosus*
- *Andryala integrifolia*
- *Crespis vesicaria* subsp*. taraxacifolia*
- *Reichardia intermedia*

Inventory 40.

- *Atractylis cancellata*
- *Leontodon longirostris*
- *Reichardia intermedia*
- *Cynodon dactylon*
